# [Retracted] MicroRNA‑616 promotes the growth and metastasis of non‑small cell lung cancer by targeting SOX7

**DOI:** 10.3892/or.2023.8623

**Published:** 2023-08-28

**Authors:** Danping Wang, Qifeng Cao, Meijun Qu, Zhaoqun Xiao, Minghui Zhang, Songbo Di

Oncol Rep 38: 2078–2086, 2017; DOI: 10.3892/or.2017.5854

Following the publication of this paper, it was drawn to the Editor's attention by a concerned reader that the cell formation assay data shown in Figs. 3D, 4D, 8D and 9D were strikingly similar to data that had already appeared in another article written by different authors at different research institutes [Wang Z, Jiang C, Chen W, Zhang G, Luo D, Cao Y Wu J, Ding Y and Liu B: Baicalein induces apoptosis and autophagy via endoplasmic reticulum stress in hepatocellular carcinoma. Biomed Res Int: 732516, 2014].

Owing to the fact that the contentious data in the above article had already been published prior to its submission to *Oncology Reports*, the Editor has decided that this paper should be retracted from the Journal. The authors were asked for an explanation to account for these concerns, but the Editorial Office did not receive a reply. The Editor apologizes to the readership for any inconvenience caused.

